# Delineating the Association between Heavy Postpartum Haemorrhage and Postpartum Depression

**DOI:** 10.1371/journal.pone.0144274

**Published:** 2016-01-25

**Authors:** Patricia Eckerdal, Natasa Kollia, Johanna Löfblad, Charlotte Hellgren, Linnea Karlsson, Ulf Högberg, Anna-Karin Wikström, Alkistis Skalkidou

**Affiliations:** 1 Department of Women´s and Children´s Health, Uppsala University, Uppsala, Sweden; 2 Department of Biostatistics, Harokopio University, Athens, Greece; 3 Department of Child Psychiatry, Turku University Hospital, Turku, Finland; 4 FinnBrain Birth Cohort Study, Turku Brain and Mind Centre, Institute of Clinical Medicine, University of Turku, Turku, Finland; St Francis Hospital, UNITED STATES

## Abstract

**Objectives:**

To explore the association between postpartum haemorrhage (PPH) and postpartum depression (PPD), taking into account the role of postpartum anaemia, delivery experience and psychiatric history.

**Methods:**

A nested cohort study (n = 446), based on two population-based cohorts in Uppsala, Sweden. Exposed individuals were defined as having a bleeding of ≥1000ml (n = 196) at delivery, and non-exposed individuals as having bleeding of <650ml (n = 250). Logistic regression models with PPD symptoms (Edinburgh Postnatal Depression scale (EPDS) score ≥ 12) as the outcome variable and PPH, anaemia, experience of delivery, mood during pregnancy and other confounders as exposure variables were undertaken. Path analysis using Structural Equation Modeling was also conducted.

**Results:**

There was no association between PPH and PPD symptoms. A positive association was shown between anaemia at discharge from the maternity ward and the development of PPD symptoms, even after controlling for plausible confounders (OR = 2.29, 95%CI = 1.15–4.58). Path analysis revealed significant roles for anaemia at discharge, negative self-reported delivery experience, depressed mood during pregnancy and postpartum stressors in increasing the risk for PPD.

**Conclusion:**

This study proposes important roles for postpartum anaemia, negative experience of delivery and mood during pregnancy in explaining the development of depressive symptoms after PPH.

## Introduction

The perinatal period is a time of intense change and transition, both in somatic and psychological modalities, leaving many women at risk for depression. Postpartum depression (PPD) often remains undiagnosed but the estimated prevalence ranges between 10–15% [1–3]. PPD has been linked to impaired mother-infant bonding and relation with the partner, and has a negative influence on the child’s emotional and cognitive development. Well known consequences for the woman herself, besides vulnerability for future affective episodes, are serious imminent ones, such as suicide in the prolonged puerperium [4–6]. Major well-known risk factors for PPD are earlier psychiatric illness, difficulties in partner relationship, low social support, socio-economic adversity, stressful life events, young age [1, 7] and problems with the infant [8].

The delivery process can become complicated, and may be experienced as traumatic [9–11]. Postpartum haemorrhage (PPH) is one of the most common obstetric complications. Despite a reduction in the number of deaths due to PPH during the last decades, it remains one of the major causes of global maternal mortality [12]. The prevalence of PPH is dependent on definitions and setting [13–15]. A common definition of PPH is a blood loss equal to or more than 500 mL, and of heavy PPH as equal to or more than 1000 mL, within 24 hours after childbirth [16]. Common estimates in the literature range between 3–6% in developed countries and the last estimate for Sweden was at 4.6% [17]. Data on the association between obstetrical complications and the risk for PPD are inconclusive [18, 19]. There are few studies that have in particular examined the association between PPH and PPD, with diverging designs, definitions, and results [20–22]. These studies have nevertheless not taken into account vulnerability in the form of earlier psychiatric morbidity or consequences of PPH. Among the most common consequences of PPH are anaemia and traumatic experience of delivery, factors that are independently associated with increased risk for PPD [23–28]. The association between PPH and PPD seems to be complex, encompassing the effects of many factors related to personal history, pregnancy and the postpartum period, such as anaemia, negative delivery experience, fatigue and breastfeeding problems.

The aim of this study was to explore the association between PPH and PPD taking into account possible confounders.

## Materials and Methods

### Study population

The women included in this nested cohort study (n = 446), were derived from two population based longitudinal studies, UPPSAT (n = 168) [29, 30] and BASIC (n = 278)[31], undertaken in the county of Uppsala investigating maternal wellbeing and depression during and after pregnancy. Uppsala is a medium sized Swedish county with a population of 323,270 inhabitants. The University Hospital is responsible for all delivering women within the county, as well as high-risk pregnancies from nearby counties. The BASIC-study is a continuation of the UPPSAT-study and the study designs are in general similar, entailing no major methodological problems with combining the two cohorts in this nested cohort study.

In the UPPSAT cohort (n = 2,318) all women giving birth at Uppsala University Hospital during May 2006 to June 2007 were asked by their midwife at antenatal care for willingness to participate. Exclusion criteria were inability to adequate communicate in Swedish, confidentially kept data, intrauterine demise or newborn in the neonatal intensive care unit. Informed consent was obtained by 65% of the target population. In the BASIC cohort (n = 2,240), all pregnant women received written information about the study in gestational week 16–18 from September 2009 to November 2012. Exclusion criteria were inability to adequate communicate in Swedish, confidentially kept data, intrauterine demise and age below 18 years. Twenty two percent of the target population has given informed consent for participation. There are no major sociodemographic differences between the two cohorts.

### Main exposure

The main exposure was heavy postpartum haemorrhage. According to clinical practice in Sweden, and also commonly used worldwide [13, 14, 32], the definition of heavy postpartum haemorrhage is considered as bleeding 1000 mL or more. This is based on the assumption that bleeding below that level is likely to be well tolerated in women without underlying medical disorders [33]. Thereby, the definition of PPH in this study was ≥1000 mL within 24 hours of delivery. The estimation of blood loss is based on inspection and/or by weighing pads and cloths and is recorded by the responsible physician or midwife in the medical records. For the purposes of this study, this estimate was extracted from the medical records.

All women in the UPPSAT and BASIC cohorts with haemorrhage equal to or more than 1000 mL within 24 hours of delivery were selected as the exposed group. Non-exposed individuals were selected by including one every 12th woman in the register of participants. To reduce misclassification, participants with bleeding between 650 and 999 mL were excluded, and the next available participant was included instead. This procedure resulted in the inclusion of 196 individuals exposed for PPH (73 from UPPSAT and 123 from BASIC) and 250 non-exposed individuals (95 from UPPSAT and 155 from BASIC).

### Outcome

The outcome was self-reported postpartum depression, defined as 12 or more points on the Swedish version of Edinburgh Postnatal Depression Scale (EPDS) [34, 35], completed by mothers in both cohorts at 6 weeks after delivery. EPDS is a self-administered instrument screening for postpartum depression. It has high validity [35, 36] and is considered the only screening tool that has enough evidence for clinical screening and for epidemiological studies in the postpartum setting. [20, 37, 38] The cut-off of 12 points is in line with the Swedish validation study [35, 39].

### Covariates

A wide range of covariates was assessed by self-reported questionnaires during pregnancy and postpartum, including socioeconomic factors, previous psychological contact (with psychologist or psychiatrist), mood during pregnancy, experience of the delivery and life situation during the postpartum period (breastfeeding, sleeping and support by partner). Data recorded by the responsible midwives and /or obstetrician on the delivery process and haemoglobin levels during pregnancy and postpartum were also available for the majority of patients.

Previous psychological contact was assessed with the question: “Have you ever been in contact with a psychologist or a psychiatrist? Yes/No”, posed at five days postpartum in the UPPSAT cohort, and in pregnancy week 17 in the BASIC cohort. Mood during pregnancy was assessed with the question: “How have you been feeling during your pregnancy?” at five days (UPPSAT) postpartum and at pregnancy week 32 in BASIC. The four alternative answers were grouped so that “Better than usual/As usual” formed one category, while “A little depressed/Depressed” formed another (Good vs. Depressed). Delivery experience was assessed in the self-report questionnaires with the item: “How would you describe your delivery experience?” with five alternative answers, posed at five days postpartum in the UPPSAT study and at six weeks postpartum in the BASIC study. The answers “Wonderful/Good/Ok” were grouped together, while the answers “Bad/Awful” were considered as suggestive of a negative self-reported delivery experience. Possible answers about breastfeeding at six weeks postpartum were “I exclusively breastfeed/ I combine breastfeeding with bottle feeding/I don´t breastfeed at all”. The two latest categories were combined (lack of exclusive breastfeeding) for the analysis. Insufficient sleep was assessed as less than an average of six hours of reported sleep per day in general, during the last few days. Partner support was assessed as insufficient if the mothers reported no or little support from their partners (vs. a lot of support).

The common definition of anaemia during pregnancy is a haemoglobin level lower than 110g/L [40, 41] and was thus also used in this study. According to the Swedish clinical guidelines, the haemoglobin level is controlled in a capillary sample at gestational week 12, week 28/29 and week 37, and recorded in the medical records by a midwife. In case of a low level, the capillary sample is usually complemented by a venous sample.

The definition of postpartum anaemia is more heterogeneous in the literature, with cut-off values ranging from 100 g/L to 120g/L [25, 40, 42–44]. The definition of postpartum anaemia (i.e. at the discharge) used in this study was 110 g/L at discharge and was extracted from the medical records. In Sweden, levels of haemoglobin directly after delivery or at discharge from the hospital are routinely obtained only in cases of PPH. A direct consequence of this practice is a high number of missing data concerning anaemia status at discharge. From earlier studies, it is known that postpartum hemodynamic changes induce a rise in haemoglobin concentrations [40]. According to that, an algorithm was produced in order to assign a value for subjects not having anaemia at discharge, even when not tested, according to the following reasoning: if the lowest haemoglobin concentration recorded during pregnancy was 105 g/L and the postpartum bleeding was less than 400 mL, the woman was classified as having no anaemia at discharge. Accordingly, women with lowest haemoglobin > 110 g/L during pregnancy are not expected to be anaemic after bleeding of <500mL. Respectively, women with lowest haemoglobin > 125 g/L during pregnancy are not expected to be anaemic after bleeding of <650mL. After insertion of values whenever possible, in accordance to this algorithm, the number of missing values haemoglobin at discharge decreased from 228 (51.1%) to 26 (5.8%).

At 6–8 weeks postpartum, the haemoglobin level should have risen to the normal level, i.e. 120 g/L or above [40]. At the same time-point, a postpartum visit is offered to the new mother, according to the Swedish guidelines. The haemoglobin levels are obtained by a capillary sample and recorded in the medical journal by the midwife. Because not all mothers attend the postpartum visit and not all are sampled for anaemia, information on the postpartum Hb levels was available only for 218 participants (51% missing values).

### Statistical analysis

Data from all participating women (i.e. socio-demographic, personal history, pregnancy and postpartum variables) were first cross-tabulated according to self-reported postpartum depression status at 6 weeks postpartum as well as PPH status. Associations were assessed with the Pearson chi-square test or Fischer’s exact test (in cases of inadequate data).

Multivariate logistic regression analysis was conducted with the EPDS-based self-reported PPD status at 6 weeks postpartum as the outcome variable, and PPH as well as possible confounders as the independent variables. The variables included as possible confounders were chosen on the basis of associations with self-reported PPD at a level of postpartum depression at a p-value of <0.1. The models were repeated with anaemia instead of PPH as the main exposure, because of the fact that anaemia could not be introduced in the earlier model due to strong co-linearity with PPH [45]. Hosmer-Lemeshow statistic was calculated to evaluate the models’ goodness-of-fit. In the logistic regression models no interactions were found statistically significant and no multicollinearity was present. All reported p-values were based on two-sided hypotheses. Statistical significance was set at a p-value of < 0.05.

Path analysis was performed, applying Structural Equation Modeling, in order to examine the proposed conceptual model i.e., the mediating effect of prenatal psychological status (previous psychological contact and mood during pregnancy), delivery related parameters (self-reported experience of delivery, baby’s birth weight), and anaemia at discharge from hospital on the association between PPH and the presence of depressive symptoms at 6 weeks postpartum. In the path analysis model the effect of postpartum stressors (lack of exclusive breastfeeding and insufficient sleep at 6 weeks postpartum) on the development of depressive symptoms was also taken into account. This approach allows for the decomposition of the total effect of PPH on depression development into direct effects and indirect effects (through each of the measured mediating risk factors).

The results of the path-analysis are shown in the estimated model where the arrows represent regression equations used to assess mediation. The strength of the relationship between two variables was estimated as a standardised regression weight (i.e., path coefficient, a). Indirect effects were obtained by multiplying the corresponding a-coefficients. Accordingly, combined indirect effects were estimated by taking the sum of the coefficients’ products for the participating pathways. While there are no established guidelines regarding sample size requirements for structural equation modelling, a generally accepted rule of thumb is that the minimum sample size should ideally be 20 times the number of variables in the model [46]. The model generated in this study consisted of 9 variables and thus the final sample of 446 was sufficient for path analysis.

Pathways indicated with a solid arrow were statistically significant (p<0.05) while dashes represent pathways with p-values from 0.05 to 0.20. The models’ goodness-of-fit was examined through the comparative fit indices (CFI and Tucker-Lewis index) which were both above the recommended level of 0.90, the standardized root mean squared residual (SRMR) which was below 0.08 and the lower bound of the 90% confidence interval of the root mean squared error of approximation (RMSEA), which was below 0.05. All of the above indices, indicated acceptable model fit [46].

IBM PASW Statistics 18.0 and STATA 12 softwares were used for the conduction of statistical analysis.

### Details of Ethics Approval

All women were informed about the course and aim of the study and gave their written informed consent. The investigation was carried out in accordance with the Declaration of Helsinki. The study protocol was approved by the Regional Research and Ethics Committee of Uppsala (UPPSAT: Dnr 2006/150, August 30, 2006, BASIC:Dnr 2009/171, July 1, 2009)

## Results

The mean age of the participating women was 31.1 years (standard deviation [SD] 4.4 years). Forty seven percent of the participants were primiparous. A total of 26% of the women had an earlier contact with psychiatrist or psychologist and 78% experienced their pregnancy as mostly positive. Twelve (3%) among the women had twins. The prevalence of preterm deliveries was 5%. At six weeks postpartum, 70% of the women were breastfeeding exclusively, 55% reported sufficient sleep (≥6 hours/day), and 58% perceived their partner as being supportive.

Table 1 presents the distribution of study subjects by PPH and a series of background, pregnancy, delivery and postpartum variables. No association with depressive symptoms at 6 weeks postpartum was noted. The association between PPH and anaemia status at discharge from the hospital was highly significant (p<0.001), while there was no association between PPH and anaemia during pregnancy or at 6 weeks postpartum. No association with sociodemographic factors as age, BMI, educational level, or parity was evident. There was a tendency of higher proportion of women with negative self-reported delivery experience among those with PPH (15% vs 9%, p = 0.101) and women with previous psychological contact (34% vs 25%, p = 0.054), although not reaching statistical significance. Significant associations were noted with mode of delivery (p< 0.001), and placental retention (p<0.001).

**Table 1:** Distribution of study participants by demographic, perinatal and clinical characteristics and association with haemorrhage at delivery.

	Haemorrhage at delivery		p-value
	< 650ml (n = 250) n (%)	≥ 1000ml (n = 196) n (%)	
Depression at 6 weeks postpartum (EPDS≥12^a^)	26 (10.4)	27 (13.8)	0.274
Anaemia at discharge from hospital (Hb^b^<110 g/L)	6 (2.6)	158 (82.7)	**<0.001**
**Background variables**			
Age (≥36 years)	31 (12.5)	32 (16.4)	0.242
BMI^c^ before pregnancy (≥30 kg/m^2^)	24 (9.8)	19 (9.7)	0.983
Education (High school or lower)	76 (34.7)	53 (28.8)	0.206
Previous psychological contact	55 (25.1)	62 (33.9)	**0.054**
**Pregnancy and Delivery related variables**			
Parity (Multipara)	131 (53.0)	103 (52.8)	0.964
Depressed mood during pregnancy	46 (22.1)	35 (20.2)	0.654
Anaemia during pregnancy (Hb <110 g /L)	58 (23.3)	48 (25.0)	0.677
Negative experience of delivery	19 (9.6)	26 (15.2)	0.101
Mode of delivery (Instrumental delivery^d^)	53 (21.2)	77 (39.3)	**<0.001**
Placental retention	1 (0.4)	61 (31.4)	**<0.001**
Severe laceration (Grade 3–4)	6 (2.4)	11 (5.6)	0.079
Preterm delivery (<37 weeks)	10 (4.1)	14 (7.1)	0.169
Infant’s birth weight (≥4 kg)	56 (22.5)	56 (28.6)	0.142
**6 weeks Postpartum variables**			
Lack of exclusive breastfeeding	65 (27.7)	52 (27.1)	0.894
Insufficient sleep (<6 hours)	102 (43.4)	78 (40.8)	0.594
Insufficient partner support	91 (39.9)	69 (36.3)	0.451
Anaemia at 6–8 weeks pp (Hb<120 g/L)	13 (8.0)	18 (12.9)	0.156

ᵃ EPDS ≥ 12 points indicates significant depressive symptoms.

ᵇ Haemoglobin

ᶜ Body Mass Index

ᵈ Instrumental delivery: Vacuum extraction or caesarean section.

Table 2 presents the distribution of women by self-reported depression (EPDS ≥ 12 points) status at 6 weeks postpartum and a series of background, pregnancy, delivery, and postpartum variables. Being exposed to PPH was not associated with depressive symptoms postpartum. There was an association with anaemia at discharge from the hospital (p = 0.014). Associations with previous psychological contact (p = 0.009) and mood during pregnancy (p<0.001) were present. A tendency of a higher proportion of women with negative experience of delivery (21% vs 11%) was noted among those depressed, not reaching significance. Use of uterotonics and/or iron supplementation did not have any association with depression status (data not shown). No exclusive breastfeeding and insufficient sleep 6 weeks postpartum were both associated with self-reported PPD (p< 0.001 and p = 0.011, respectively).

**Table 2:** Distribution of study participants by demographic, perinatal and clinical characteristics and association with self-reported depression status at 6 weeks postpartum.

	Depression at 6 weeks postpartum		p-value
	EPDS^a^: 0–11 (n = 393) n (%)	EPDS: 12–30 (n = 53) n (%)	
Haemorrhage (<1000ml)	169 (43.0)	27 (50.9)	0.274
Anaemia at discharge from hospital (Hb^b^<110 g/L)	137 (36.9)	27 (55.1)	**0.014**
**Background variables**			
Age (≥36 years)	55 (14.1)	8 (15.1)	0.846
BMI^c^ before pregnancy (≥30 kg/m^2^)	35 (9.0)	8 (15.4)	0.143
Education (High school or lower)	115 (32.2)	14 (30.4)	0.808
Previous psychological contact	96 (27.0)	21 (45.7)	**0.009**
**Pregnancy and Delivery related variables**			
Parity (Multipara)	206 (53.0)	28 (52.8)	0.986
Depressed mood during pregnancy	62 (18.3)	19 (44.2)	**<0.001**
Anaemia during pregnancy (Hb <110 g /L)	88 (22.6)	18 (34.6)	**0.057**
Negative experience of delivery	36 (11.0)	9 (21.4)	**0.052**
Mode of delivery (Instrumental delivery^d^)	114 (29.0)	16 (30.2)	0.859
Placental retention	54 (14.1)	8 (15.1)	0.846
Severe laceration (Grade 3–4)	16 (4.1)	1 (1.9)	0.706
Preterm delivery (<37 weeks)	21 (5.5)	3 (5.7)	0.999
Infant’s birth weight (≥4 kg)	96 (24.5)	16 (30.2)	0.370
Haemorrhage * Anaemia at discharge			**0.007**
No Haemorrhage/No Anaemia	203 (54.7)	20 (40.8)	
Haemorrhage/No Anaemia	31 (8.4)	2 (4.1)	
No Haemorrhage/Anaemia	3 (0.8)	3 (6.1)	
Haemorrhage/Anaemia	134 (36.1)	24 (49.0)	
**6 weeks Postpartum variables**			
Lack of exclusive breastfeeding	92 (24.5)	25 (48.1)	**<0.001**
Insufficient sleep (<6 hours)	149 (39.9)	31 (58.5)	0.011
Insufficient partner support	137 (37.4)	23 (44.2)	0.345
Anaemia at 6–8 weeks pp (Hb<120 g/L)	27 (10.2)	4 (10.5)	0.999

ᵃ EPDS ≥ 12 points indicates significant depressive symptoms.

ᵇ Haemoglobin

ᶜ Body Mass Index

ᵈ Instrumental delivery: Vacuum extraction or caesarean section.

Compared to women without previous psychological contact and PPH, women with both these exposures had higher OR (OR = 3.71; 95% CI 1.65–8.35, p = 0.002) for developing PPD than women with either previous psychological contact or PPH alone.

Table 3 presents multivariate logistic regression derived ORs and 95% CIs for significant depressive symptoms, adjusted for previous psychological contact, experience of delivery, mood during pregnancy and no exclusive breastfeeding at 6 weeks postpartum. While there was no statistically significant association in regards to PPH, anaemia at discharge was associated with higher risk for significant depressive symptoms postpartum (adjusted OR = 2.29, 95%CI = 1.15–4.58). Interaction terms between PPH and anaemia, respectively, and possible confounders were not statistically significant (data not shown).

**Table 3:** Multivariate logistic regression derived odds ratios (ORs) and 95% Confidence Interval (95% CI) for self-reported depression status (EPDS≥12) at 6 weeks postpartum.

	Model 1^a^ OR (95% CI)	Model 2^b^ OR (95% CI)
Haemorrhage (ml) (≥1000 *vs* ≤650)	1.81 (0.91–3.57)	
Anaemia at discharge from hospital (Hb<110 g/L vs Hb≥ 110 g/L)		2.29 (1.15–4.58)
Previous psychological contact	2.08 (1.05–4.10)	1.90 (0.95–3.80)
Mood during pregnancy (Depressed *vs* very good/ok)	3.02 (1.51–6.06)	2.87 (1.40–5.87)
Lack of exclusive breastfeeding at 6 weeks pp	2.30 (1.16–4.56)	2.41 (1.20–4.85)

^a^ Model 1 includes PPH and possible confounders as independent variables.

^b^ Model 2 includes anaemia and possible confounders as independent variables.

**Fig 1:**
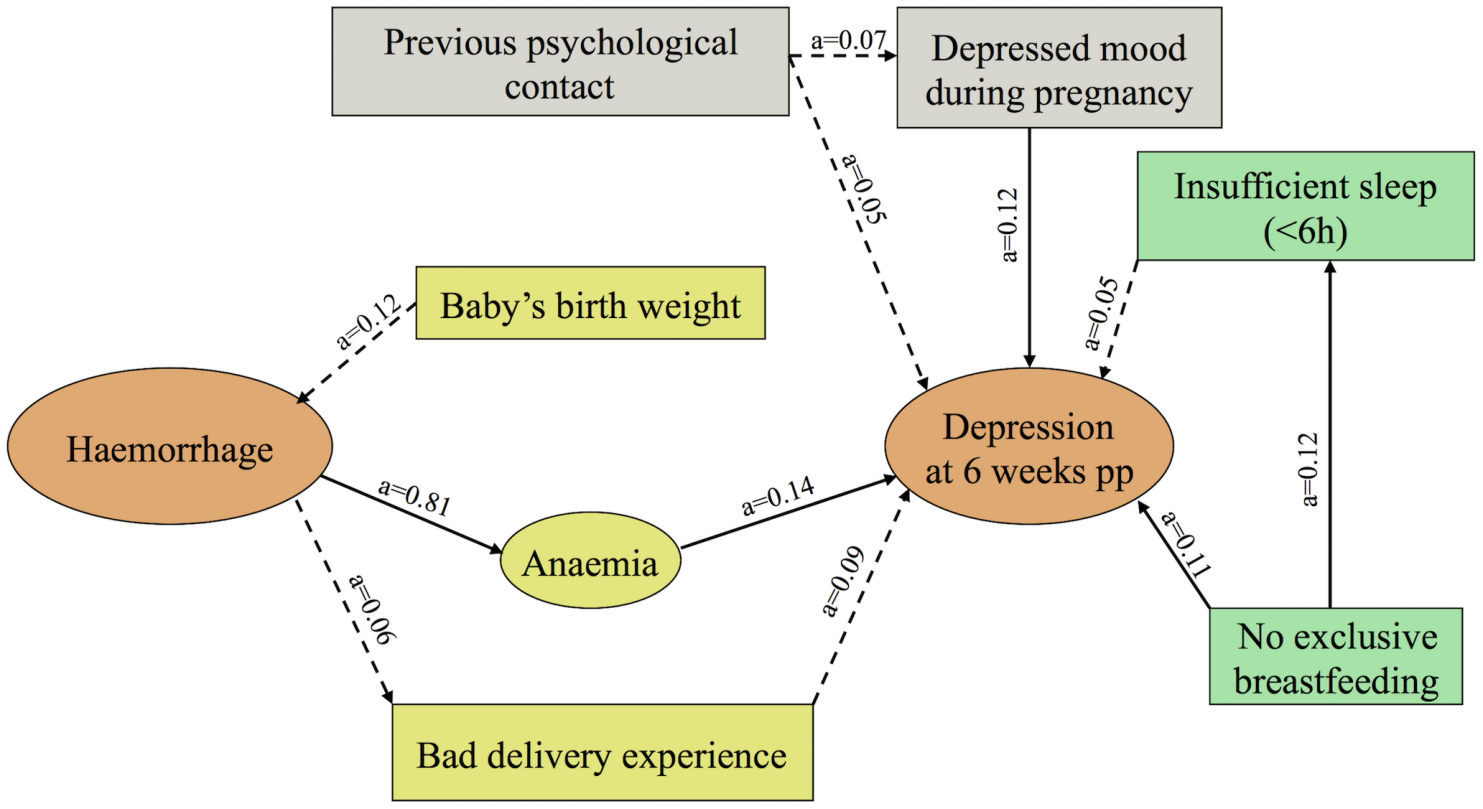
Graphic display of significant pathways associated with self-reported depression status 6 weeks postpartum. Graphic display of the significant pathways through which postpartum haemorrhage (PPH) and other delivery related variables as well as earlier psychological contact, lack of exclusive breastfeeding and inadequate sleep at 6 weeks postpartum influence depression status at 6 weeks postpartum. Pathways indicated with a continuous arrow were statistically significant (p<0.05). Dotted arrows represent pathways with p = 0.05–0.20.

Results from the path analysis are presented in Fig 1. Direct and positive influences on PPD were observed for previous psychological contact (a = 0.05), depressed mood during pregnancy (a = 0.12), anaemia at discharge from hospital (a = 0.14), self-reported bad delivery experience (a = 0.09), lack of exclusive breastfeeding (a = 0.11,) and insufficient sleep (a = 0.05) at six weeks postpartum. The infant´s birth weight did not have a statistically significant direct or indirect impact on PPD but influenced the risk for PPH (a = 0.12), indirectly affecting the development of anaemia (a = 0.09). PPH did not have a direct effect on PPD but indirectly increased the risk for PPD through anaemia and bad delivery experience (a = 0.12). Furthermore, previous psychological contact influenced depressed mood during pregnancy (a = 0.07), indirectly affecting depression status (a = 0.01). Finally, lack of exclusive breastfeeding had an impact on insufficient sleep (a = 0.12) but its indirect pathway to PPD, through insufficient sleep, was not significant.

## Discussion

### Main Findings

In this study, postpartum depressive symptoms were not found to be associated with PPH per se, but rather with anaemia at discharge from the maternity ward, mood during pregnancy and negative experience of delivery. The path-analysis used in this study provided insight in the complex mediating roles of several consequences of postpartum haemorrhage, such as anaemia and negative self-reported delivery experience, and postpartum stressors (insufficient sleep and no exclusive breastfeeding) in shaping the risk for PPD.

Well-known associations of PPH and instrumental deliveries and placental retention were replicated in the current study. However, other well-known risk factors as obesity and multiparity delivery were not identified in this material, probably due to both power issues and the cohort's composition, with a small number of women with high BMI and no women with grand-multiparity [12, 13, 47]. The same might apply for preterm delivery, due to the fact that infants admitted to the neonatal intensive care united were excluded from participation in the UPPSAT study. In accordance with the literature, the associations of postpartum depression with previous psychological contact, mood during pregnancy, insufficient sleep and breastfeeding problems were statistically significant in this study. Associations with other risk factors such as educational level and partner support were nevertheless not replicated [1, 7]. A possible explanation is an over-representation of well-educated, healthy women in the cohorts of our study.

The results of this study can be explained in terms of pathophysiological mechanisms. A major somatic consequence of PPH is anaemia, which presents with a symptomatology including fatigue, reduced cognitive abilities, emotional instability, and subsequently depression [40, 48, 49]. Psychological consequences of PPH include postpartum distress symptoms [11] and a negative experience of delivery, which have been linked with increased risk for postpartum depression [50, 51]. Beyond that, there is a synergistic effect between some of the above mentioned parameters, such as earlier psychological contact and PPH. The path analysis illustrates the relative contributions of earlier psychological contact and mood during pregnancy, delivery-related complications and their consequences such as anaemia and negative self-reported delivery experience, and lastly postpartum variables, such as breastfeeding and sleep, in the development of depressive symptoms postpartum.

The few studies examining the association between PPH and PPD have diverging methods and results. A Jordanian study by Mohammad et al, found an association with PPD at 6–8 weeks postpartum but not at six months [20]. No information was given on either the haemoglobin levels or whether transfusions were given. On the contrary, a multi-centre study from Australia did not demonstrate any effect of PPH on either emotional or physical health outcomes [21]. However, the majority of women in the Australian study did receive a transfusion, which probably compensated for the blood-loss. Presumably, the prevalence of anaemia at discharge was thereby higher in the Jordanian study. Mohammad et al also stress the association of dissatisfaction with the delivery and development of PPD, in accordance with the current study. A retrospective study on severe PPH with embolization found that severe PPH may have long-term psychological impact on women, but did not study PPD in particular [22].

The above mentioned studies have nevertheless not taken into account consequences of PPH; anaemia, most commonly, and the traumatic experience itself. These factors have been associated with depression in general and should therefore be considered when assessing the association between PPH and PPD. Earlier studies focusing on anaemia and PPD are heterogeneous. Some identified postpartum anaemia as a significant risk factor for the development of PPD [23–27]. Other studies report no association [37] or even a negative association between anaemia and depression during pregnancy [52]. The path-analysis used in the current study can thereby clarify earlier diverging studies about PPH and PPD, while replicating earlier study results on the association of PPD, distress during pregnancy [53, 54], negative self-reported delivery experience, breastfeeding and sleep postpartum [55–57], as and anaemia at discharge [20, 22]

It is noteworthy that despite the potential serious outcomes of postpartum anaemia, the optimal treatment strategies are still unclear, both regarding to chosen curative dose, timing and criteria [3, 58]. However, most studies and guidelines agree on the restrictive use of red blood cell transfusions in favour of iron (oral or intravenous), while stressing the importance of taking into account side effects, available resources and each individual woman’s symptoms and comorbidities [3, 15, 58–60].

### Strengths and Limitations

The study population is composed from two different cohorts (UPPSAT and BASIC). UPPSAT had a higher participation rate and was more representative of the background population [30]. A plausible reason for the lower participation rate in the BASIC study is the comprehensive nature of the study, including not only multiple questionnaires but also an extended sampling of biological samples with the possibility of genetic analyses. Mothers in the BASIC cohort had in general a higher education than those in the UPPSAT cohort, a variable that was not associated with either PPH or PPD, and rates of PPH were comparable in the two cohorts.

The lower participation rate is not expected to greatly influence associations reported in this material, but could limit the generalizability of the findings. Furthermore, nearly equal numbers of exposed and non-exposed individuals were selected from both samples, so differences in some response variables would not be expected to influence the results. The exclusion of individuals with bleeding 650–999 mL, decided on in order to reduce misclassification and increase the power of the study, could theoretically affect the generalizability of the results.

There were some small differences in the time-points when some of the variables were assessed but there was no great difference in the prevalence of the different response alternatives. A possible limitation of the study is the need for the creation of an algorithm in order to reduce the proportion of missing data on anaemia at discharge, caused by current clinical practice advising for obtaining haemoglobin concentration postpartum only after a subjective heavy bleeding. Nevertheless, the use of this strict algorithm entails very low risk of misclassification and is not expected to have introduced any bias. Other limitations were the small number of individuals in certain categories, such as preterm, twin deliveries and grand multipara, and the absence of information on medication with selective serotonin re-update inhibitors (SSRIs) and bleeding disorders. The outcome variable, depression status, was assessed through a self-reported scale, the EPDS, and not a psychiatric interview. The EPDS is an instrument used for screening purposes with different cut-offs for screening for depression, ranging in the literature between 10 and 13 [20, 37, 38]. The cut-off used in this study is 12 points, in line with the Swedish validation study, which states a sensitivity of 96% and a specificity of 49% [61]. Our results therefore apply to significant depressive symptoms, and not clinical depressive episodes, but in accordance with the literature, EPDS has a high sensitivity and specificity [39]. This study was the first one to use a path analysis approach in examining the association of heavy postpartum bleeding and depressive symptoms postpartum, and its results should be replicated even in different settings.

On the other hand, the use of two longitudinal, population-based cohorts in Sweden, and the availability of information on the majority of the variables of interest in this context are among the strengths of this study. The cohort's sociodemographic distribution corresponds well with the Swedish statistics (the Swedish Birth Register), except for the educational level, which was higher in our nested cohort (61% with university education vs. 52% in the Swedish Birth Register)[62]. A further advantage of the current study is the implementation of two different statistical methods, with convergent final results, in order to assess the association between PPH and depressive symptoms postpartum.

## Conclusion

This study proposes that clinicians should carefully monitor postpartum anaemia and consider active treatment after heavy postpartum bleeding to reverse the anaemia. The importance of screening of depressive symptoms and of timely interventions aiming at preventing an unresolved negative self-reported delivery experience is also stressed. These results, coming from the first study using a path-analysis approach to tackle the complexity of the studied situation, stress the importance of primary and secondary prevention of postpartum anaemia, and of offering psychological support to women with negative self-reported delivery experience and history of psychological conditions.

## Supporting Information

S1 Dataset (SAV)
